# Denture impaction in the oesophagus experience of a young ENT practice in Nigeria

**DOI:** 10.11604/pamj.2014.18.330.2871

**Published:** 2014-08-25

**Authors:** Taiwo Olugbemiga Adedeji, Adedayo Olugbenga Olaosun, Olusola Ayodele Sogebi, James E Tobih

**Affiliations:** 1 Department of Otorhinolaryngology Head and Neck Surgery, LAUTECH Teaching Hospital, Osogbo, Osun State, Nigeria; 2 Department of Surgery (ENT), College of Health Sciences, Olabisi Onabanjo University, Sagamu, Nigeria

**Keywords:** Oesophageal dental impaction, Acrylic dentures, complications, Nigeria

## Abstract

**Introduction:**

The effect of dental loss and associated desire to restore its function and aesthesis has led to an increase in the number of people wearing dentures. This study therefore reviews the cases of impacted acrylic dentures in the oesophagus.

**Methods:**

A retrospective review of patients that were managed for oesophageal denture impaction at Ladoke Akintola University of Technology Teaching Hospital, Osogbo, Osun State, Nigeria, over an eight year period from 2005 to 2012.

**Results:**

A total of 14 patients (M: F 2.5: 1). The age ranged from 32 - 75 years. Majority 64.3%) were 51 years and above. Over 70% presented early. Major presenting symptoms were throat pain (100%), odynophageal (92.9%) and dysphageal (78.6%). The radiographic findings were air entrapment (64.3%) and increase in prevertebral soft tissue shadow (78.6%). Majority (87.5%) were impacted at the upper (cervical) oesophagus. Over 78% had successful extraction with rigid oesophagoscopy. Two (14.3%) had spontaneous expulsion of the denture and 1 (7.1%) discharged himself against medical advice. Complications were mucosa tear (28.6%), laryngeal spasm/ airway obstruction (14.3%), mucosa oedema/ erythema (57.1%), neck abscess (7.1%).

**Conclusion:**

Impaction of esophageal dentures is relatively common in our locality; most patients present early for medical attention and associated with successful rigid oesophagoscopies and denture extraction under GA, and generally good outcome. Education of the wearers of dentures was emphasized as a way of preventing dentures impaction.

## Introduction

Missing teeth from any cause in humans leave gaps within the gums which is not aesthetically appealing [1, 2]. Leaving missing teeth untreated can accentuate oral trauma, discomfort and facial sinking or sagging[3] and people with missing teeth often experience a lack of confidence in their appearance[1, 3]. When teeth are missing, chewing ability is impaired. Missing teeth affect food choices, digestion and healthy nutrition intake. Replacement of missing teeth is therefore highly desirable in order to restore the defect and regain function as best as possible[2] Acrylic dentures are removable teeth replacement options which are made and fabricated from poly - methylmethacrylate a radiolucent material. They are designed as substitute teeth to fill gaps in gum left by missing teeth. Acrylic dentures are simple in design and cost-effective, made to fit and support oral function and facial expression [1, 2]. They also allow patients to chew food and engage in communication with other people like any normal person by facilitating normal communication without pronunciation difficulties. Thus they strengthen speech and communication with lowering the risk of misunderstandings and communication break-downs[2–3]. It is therefore observed that people with missing teeth who wear dentures experience improved health and quality of life, than those who do not [4–7]. The effect of dental loss and associated desire to restore its function and aesthesis has led to an increase in the number of people wearing dentures [7–10]. This was stated by Okeowo et al [5] in Nigeria over two decades ago. Tihan et al. [11] in United States of America reported that 1 out of 5 persons aged 18 - 74 years have partial or total dental prosthesis.

Despite being very useful, denture is not without problems and complications. Minor complaints like the feeling of wearing of foreign body, loss of sensitivity of the gum in the area of insertion of the dentures have been reported [12, 13]. However, almost a third of edentulous patients wearing dentures had their major complaints bothering on stability and retention of these “foreign bodies”. The most dreaded complication of dislodgement of dentures is impaction in the esophagus! The consequence of increasing number of people that are wearing dentures has led to a proportionate increase in the incidence of esophageal impacted dentures [4–6] with its associated problems [10–12]. In Nigeria reported prevalence of impaction of acrylic dentures in the esophagus ranges between 1.3% - 38.6% [4, 5, 7, 14]. Factors predisposing to dentures dislodgement from the gums and impaction into the esophagus include inappropriate fabrication, prolonged usage, and failure of patients in keeping appointments for dental clinic follow-up evaluations especially when the denture becomes unstable [4]. Furthermore, acrylic dentures are associated with reduced sensitivity of the oral cavity because of the insulating nature of the resins used in their fabrication [8, 9]. However, many of the impactions are accidental in nature. Presentation of esophageal impacted dentures is often straightforward but dramatic, and urgent and adequate management of patients will need to be instituted. The simplest form of intervention is removal under direct vision via rigid Oesophagoscopy [4]. However removal of difficult impactions via cervical oesophagotomies has been reported [4]. Delayed intervention is associated with complications which can increase the morbidity of the patient. Rarely impacted oesophageal dentures can be fatal. There have been scanty reports on oesophageal dentures impaction from relatively bigger ENT centers in the country, this study is a review of the cases of impacted acrylic dentures in the oesophagus, in a relatively young ENT practice in a sub-urban center of a tertiary health facility. It assesses the clinical and radiological presentation, as well as the management and outcome of patients. The study also noted if there are major differences between these and what were reported from the other centers.

## Methods

The study was a retrospective review of all patients that were admitted and managed for denture impaction in the Oesophagus at the Ear, Nose and Throat department of Ladoke Akintola University of Technology Teaching Hospital, Osogbo, Osun State, Nigeria, over an eight year period between 2005 and 2012. The medical records of the patients were retrieved from the case notes through the medical records department of the hospital. Data generated with retrieval of information which included patients’ age, sex, symptoms at presentation, occupation, mode of denture impaction, duration of symptoms before presentation elicited clinical signs, radiographic findings, duration between before presentation, duration between presentation and surgical intervention, level of denture impaction in the oesophagus, status of the esophageal mucosa after denture extraction, outcome of intervention, duration of hospital stay after surgical intervention, and final outcome. Excluded were patients whose case records could not be located and those that had missing significant and vital information The information was entered into a spread sheet and the data generated was analysed using SPSS version 14 (Chicago, IL). The data was presented in simple descriptive forms as proportions using tables and graphic chart.

## Results

A total of 19 patients were admitted and managed for oesophageal denture impaction during the study period. Out of these 14 patients had their complete record retrieved for data analysis. This represents 18.4% of 76 patients that had various pharyngo-oesophageal foreign bodies during the period of study. There were 10 (71.4%) males and 4 (28.6%) females with ratio 2.5: 1 (M: F). The age of the patients ranged from 32 - 75 years, the modal age group is 61 - 70 years (28.6%) with a mean age of 56.07 (SD ± 14.73). Table 1 shows age, sex and occupation distribution of the patients. Almost two-thirds (64.3%) of the affected were above 51 years in age. Throat pain was the most common symptom that was present in all the affected patients. Other symptoms and signs, duration before presentation at the hospital and mechanisms of injury were as shown in Table 2 below All the patients had plain lateral soft tissue radiograph of the neck to confirm the diagnosis while 3 (21.4%) patients had additional barium swallow done to determine the level of impaction. The main radiographic findings and the levels of denture impaction at rigid oesophagoscopy were as shown in Table 2 below. Eleven patients (78.6%) had successful extraction of the impacted denture with rigid oesophagoscopy. Two (14.3%) had spontaneous expulsion of the denture (via the anus) and 1 (7.1%) discharged himself against medical advice. One patient initially had failed attempted removal and on second attempt had denture fragmented by shear forceps before extraction and the denture was removed in pieces.

**Table 1:** Socio-demographic characteristics of the patients

Variable		
Age ( Years)	Frequency	Percentage
31-40	3	21.4
41-50	2	14.3
51-60	3	21.4
61-70	4	28.6
71-80	2	14.3
Mean ±SD		
**Sex**	56.7±14.7	
Male	10	71.4
Female		
**Occupation**	4	28.6
Trading	5	35.7
Farming	4	28.6
Civil servant	2	14.3
Artisan	1	7.1
Clergy	1	7.1
Dependant	1	7.1

**Table 2:** Clinical characteristics of the patients

Variable	Frequency	Percentage
**Symptoms and sign**		
Throat pain	14	100.0
Odynophagia	13	92.9
Dysphagia	11	78.6
Drooling/pooling of saliva	5	35.7
**Duration of symptoms (hours)**		
0-24	10	71.4
>24	4	28.6
**Mechanism of injury**		
Accidental	14	100.0
Deliberate self-harm	nil	0.0
**Radiological findings**		
Air entrapment in oesophagus	9	64.3
Increase pre-vertebral soft tissue	11	78.6
**Level of impaction (at oesophagoscopy)**		
Upper	12	85.7
Middle	2	14.3
Lower	nil	0.0

Note: Twelve patients (85.7%) had ≥2 symptoms/signs

Various complications associated with denture impaction and its management, outcome of treatment and duration of hospital stay were as shown in Table 3 below. Two patients with airway distress postoperatively had emergency tracheostomies; the patient with neck abscess had incision and drainage of the abscess and placed on broad spectrum antibiotics, while those with mucosa tear were successfully managed conservatively with nasogastric tube feeding and broad spectrum antibiotics and analgesics. All the patients except only one that discharged against medical advice were successfully managed and discharged home and no mortality was recorded.

**Table 3:** Post intervention assessment-complications, duration of hospital stay and outcome

Variable		
Complication	Frequency	Percentage
Oesophageal mucosa tear	4	28.6
Laryngeal spasm/airway obstruction	2	14.3
Erythema/oedema	8	57.1
Neck abscess		
**Duration of hospital stay (weeks)**	1	7.1
<1	9	64.3
1-2	3	21.4
>2		
**Outcome**	2	14.3
Fully recovered and discharged home	13	92.9
Discharged against medical advice	1	7.1

Note: Some patients had more than one complication

## Discussion

There is a risk of oesophageal denture impaction with the use of acrylic dentures. Such impaction poses a challenge to the patients and to the medical personnel due to its associated high complication rate [10–17]. Its management modalities associated morbidities and preventive measures will need to be addressed. Male gender is generally more predisposed than the female gender. This fact was supported by the present study with 71.4% prevalence in male. This was corroborated by findings from previous studies [4, 15, 16]. Agrawa et al [16] reported male preponderance of 86%. Although greater proportion of females compared to males may be wearing dentures for cosmetic reasons; it appears that females are more conscious of their health and tend to take care of their dentures better than males through regular medical checkup [4]. Smoking is also prevalent among males with its associated nicotine induced poor mucosal sensations [15]. This may also contribute to higher prevalence of denture impaction among males. The higher prevalence (64.3%) of denture impaction among the traders and farmers seen in this study, could this be a reflection of their high level of ignorance, poverty, carelessness in handling the dentures and failure to present for routine medical checkup even when they have noticed their denture is loose or shaking The sample size for this study is however small to justify this assertion and a more extensive, controlled study which will incorporate level of education and possibly knowledge and attitudes of the patients, is suggested.

As reported by other researchers [2, 10–17], all our patients swallowed their dentures accidentally. Dentures are worn more by adults compared with children, who could be more careless in their handling. All our patients in this study were adults, however due to the previously mentioned factors; dentures could dislodge and be swallowed accidentally in adults. On the other hand, denture impactions for deliberate self harm [12], or also in unconscious and subconscious or in people with learning difficulty have been reported [12, 13] The presentation of denture impaction is often dramatic but the diagnosis is straight-forward many times. However, it may sometimes pose a diagnostic challenge [11–13, 16] and at such instances high index of suspicion will be required by both the denture wearer and the clinician. A diagnostic failure of impacted dentures of up to 47% has been reported [12] by Hashmi et al in UK. Sudden throat pain, after swallowing food, drink or drugs, in a person that wears dentures is the most common presenting symptom found in this study [4, 16]. Singh et al [17] and Tihan [11] however reported dysphagia as the most presenting symptom in their patients. Throat pain and dysphagia are actually similar and it may be difficult to differentiate between them unless a person has had some medical training, thus both can be used synonymously by patients. Plain radiographs remains the most basic investigation for patients with suspected impacted esophageal dentures, since this will confirm the diagnosis, give an idea of the level of impaction, and possibly detect complication like esophageal perforation. Acrylic dentures seen in all the cases in the present study were made of poly (methylmethacrylate) a radiolucent material, which may be difficult to detect with a standard plain radiograph [12], thus in doing plain radiographs for dentures will necessitate a soft tissue exposure. Radiological diagnosis in this type of oesophageal foreign bodies require experience and air entrapment and increase in prevertebral soft tissue shadow is usually suggestive of denture impaction as observed in the majority of the cases in this study. Previous studies have also reported similar findings [4, 12, 18, 19]. In order to allow easier localization of dentures using plain X rays, some researchers had suggested incorporation of metal pins/clasp that attaches the teeth to the denture base and incorporation of radio-opaque material to the denture [12, 15–18]. Alternatively, the use of barium (a contrast material) to aid localization of level of denture impaction in those cases that were unclear has been reported [4, 16] and three of the patients in this study had this investigative procedure used to localize the impacted dentures. The major limitation to the use of barium contrast medium is the coating of all the sides of radiolucent object [12] and it should therefore not be performed just prior to an endoscopy of the upper GIT [12]. Computerized tomographic scan (CT scan) and magnetic resonance imaging (MRI) are other investigations of choice that aid localization of impacted denture in cases that are difficult to diagnose[12, 16, 20, 21]. While foreign body impaction in the esophagus occur at the three anatomic areas of constriction, the site of denture impaction usually at the first area of constriction in the upper third of the oesophagus just below the cricopharyngeal junction [4, 14, 15, 16]. This is attributable to the rather peculiar nature of the denture as a foreign body which has serrated edges, and has a wire clasp for stabilization and also because of the relatively big size. All these factors predispose to the impaction at the first area of constriction and most of them may not slip further down the esophagus [4, 11, 15]. 85% of the cases of dentures in this study got impacted at the upper oesophagus the remaining 15% got impacted at the middle oesophagus. None of the impacted denture was found at lower oesophagus in this study. Rarely, some dentures got impacted at the lower third of the esophagus, and these are associated with more difficulty in removal, as well as more complications [17, 22–24].

Once diagnosis is confirmed, the treatment is immediate removal of the denture from the oesophagus, there is no room for conservative management [11, 12, 15, 19] and delay in removal predisposes to more complications [10–17]. Some of these complications include local necrosis of the mucosa at the site of impaction, formation of fistulae and vascular erosion with excessive haemorrhage which may be fatal [4, 17]. Extraluminal migration with subsequent development of a diverticulum has also been reported following delayed removal of impacted denture [12]. while Akinpelu et al. [10] reported a case of Horner´s syndrome that developed in a 26-year-old woman following accidental swallowing of an upper denture which then became impacted in the oesophagus, the syndrome abated completely by the seventh day post-surgery following denture extraction. It is obvious that immediate and urgent extraction of impacted oesophageal denture is non-negotiable. This is achieved by performing a rigid oesophagoscopy using a scope with the widest possible diameter for the patient [17, 19], and denture extraction under general anesthesia. [11, 12, 17, 19]. This will facilitate proper illumination and visualization. This method of removal will also facilitate placement of the denture inside the lumen of a wider rigid oesophagoscope before its withdrawal which will therefore protect the mucosa from the sharp and metallic end of the denture. This manoevre will not be possible using a flexible oesophagoscope because, chances of oesophageal perforation would be much higher if the flexible endoscope is used for the removal of voluminous or sharp foreign bodies like dentures [17]. All the dentures in this study were extracted using the rigid oesophagoscope. Singh et al. [17] reported a case of an iatrogenic oesophageal perforation with denture which occurred when attempted removal with flexible oesophagoscopy was made. All precautions should be taken during denture removal and attention must be paid to details of the operative technique to prevent catastrophic oesophageal perforation[17] The procedure should not be done hastily, over enthusiastic manoevre should be avoided, while instrumentation should be gentle and meticulous. Most (78.6%) of our patients had successful removal with rigid oesophagoscopy. This agreed with the previous studies [4, 15, 17]. Extraction of difficult cases can be facilitated by the use of shears forceps for fragmentation of denture to ease extraction [4, 17] as was the case with one of our patients. Other methods of denture removal in difficult and complicated cases include extraction via either transcervical [4, 10, 15] or transthoracic oesophagotomy [11, 15, 16]. Two of our patients had spontaneous expulsion per via naturalis when the dentures passed down and got out with the stool. This situation has also been previously reported by Mishra et al [15]. Such occurrences however occur sporadically and do not justify expectant, watchful or conservative management. Care should also be taken during extraction process to avoid advancing the scope beyond the denture even if the impaction is close to the oesophagogastric junction [17]. This has a tendency to dislodge and translocate the denture into the soft tissue space or cause an iatrogenic oesophageal perforation. Tihan et al. [11] reported an iatrogenic oesophageal perforation by the hook of the denture when impacted denture was been pushed down to the stomach for manipulation and extraction.

Another factor that may determine the outcome of impacted dentures is the time of presentation for medical attention. Baring other factors, early presentation leads to a good outcome, and vice-versa. Incidentally, some of the patients because of cultural, social and religious reasons, belief and patronize unorthodox medical practitioners, before presenting at the medical facilities. Furthermore, accessibility and affordability of the medical care and availability of the needed medical expertise may also influence time of presentation and patronage of the facilities.71.4% of the patients presented to the hospital within 24 hours of impaction possibly because the hospital is located right within the heart of the town and easily accessible to the populace. In some of unorthodox medical facilities, unconventional maneuvers and manipulations are done often without success, only for the patients to present to the Otolarynogologists after complications had set in. However, complications can also occur from conventional rigid oesophagoscopy. Over 85% of our patients had various complications with mucosa erythema/ oedeme (57.1%) and mucosa tear (28.6%) been the common complications observed. Two patients that developed acute airway obstruction following denture extraction in this study might have resulted from laryngeal spasm as a result of manipulation of the impacted denture around the cricopharyngeal junction. Acute laryngeal oedema may also result following such manipulation with subsequent airway compromise. Denture itself may get impacted in the airway with subsequent respiratory distress, with the patient presenting in acute upper airway obstruction. In such a situation securing the airway becomes the priority and first line of management. The two patients in this study however developed respiratory distress after the dentures have been extracted and were rescued with emergency tracheostomies. Other complications observed were successfully managed with incision and drainage, broad spectrum antibiotics, nasogatric tube feeding and analgesic. It is vitally important that every doctor that is performing a surgical procedure be conversant with recognition and management of common complications associated with the procedure.

Complications are associated with increased morbidity, prolonged hospital stay and high cost, loss of revenue and occasionally mortalities. These issues are more germaine in resource poor developing countries of sub-saharan Africa like Nigeria. However, impaction of oesophageal dentures is highly preventable with the proper education and motivation of the denture wearer. Denture wearers need to be educated on predisposition to, risk of impaction, denture life span, denture maintenance, need to keep up regular visit appointment for retention assessment. Also the importance of immediate presentation at the hospital once dislodgement and impaction has occurred should be stressed to them. Duration of hospital stay in our patients ranged from three days to nineteen days, generally longer hospital stay were noted in those with major complications like neck abscess, mucosa tear and those with tracheostomies due to respiratory distress. Our observation was that the patient with neck abscess presented late when infection had set in. The fact that no mortality was recorded from this study may be a reflection that majority of our patients presented early and that there was prompt and urgent intervention. The need for educating denture wearer for denture maintenance and early presentation following impaction cannot be therefore over emphasized.

## Conclusion

In conclusion, impaction of esophageal dentures is relatively common in our locality, most patients present early for medical attention, diagnosis confirmed with plain x-ray soft tissue neck, associated with successful rigid oesophagoscopies and denture extraction under GA, and generally good outcome. Education of the wearers of dentures was emphasized as a way of preventing dentures impaction.
